# Cost-effectiveness of anatomic total shoulder arthroplasty versus pyrocarbon hemiarthroplasty for glenohumeral osteoarthritis: a 10-year Markov cost-utility analysis

**DOI:** 10.1016/j.jsea.2026.100094

**Published:** 2026-09-23

**Authors:** Akin A. Adio, Andrew Bouras, Joseph A. Abboud, Grant E. Garrigues, Robert J. Gillespie, Thibault Lafosse, William N. Levine, Frederick A. Matsen, Hafiz F. Kassam

**Affiliations:** a Perelman School of Medicine, University of Pennsylvania, Philadelphia, PA, USA; b Nova Southeastern University, Dr. Kiran C. Patel College of Osteopathic Medicine, Clearwater, FL, USA; c Division of Shoulder and Elbow Surgery, Rothman Orthopaedic Institute, Philadelphia, PA, USA; d Midwest Orthopedics, Rush University Medical Center, Chicago, IL, USA; e University Hospitals Drusinsky Sports Medicine Institute, Cleveland, USA; f Alps Surgery Institute, Clinique Générale Annecy, Annecy, France; g Department of Orthopedics, New-York Presbyterian/Columbia University Irving Medical Center, New York, USA; h Department of Orthopaedics and Sports Medicine, University of Washington Medical Center, Seattle, WA, USA; i Hoag Orthopaedic Institute, Irvine, CA, USA

**Keywords:** Pyrocarbon hemiarthroplasty, Hybrid glenoid, All-polyethylene glenoid, Anatomic total shoulder arthroplasty, Cost-effectiveness, Markov model, Shoulder osteoarthritis

## Abstract

**Background:**

Pyrocarbon hemiarthroplasty (HA) is used for glenohumeral osteoarthritis in younger patients to preserve glenoid bone, but its cost-effectiveness against anatomic total shoulder arthroplasty (aTSA) has not been evaluated.

**Methods:**

A Markov cost-utility model compared Tornier pyrocarbon HA, hybrid aTSA, and all-polyethylene aTSA in a 65-year-old over 10 years from a US hospital perspective (2026 dollars, 3% discount). Well-state utilities were equalized and a revision-year decrement was applied. Threshold, one-way, scenario, and probabilistic analyses were performed, including younger-patient scenarios using age-specific registry aTSA revision rates.

**Results:**

Hybrid aTSA dominated pyrocarbon HA (−$2,332; +0.015 quality-adjusted life years [QALYs]), as did all-polyethylene aTSA (−$1,633; +0.009 QALYs), with 84% and 75% probability of cost-effectiveness at $50,000 per QALY. Pyrocarbon HA became cost-effective only if its 10-year revision rate fell from 10.6% to 1.0% or 4.2% or its cost fell by $3,066 or $2,063. aTSA lost its advantage when its own 10-year revision rate exceeded 12.8%. In patients under 65, aTSA remained marginally cost-effective with modern-prosthesis registry revision rates (10.5% at 10 years; incremental net monetary benefit $711) and pyrocarbon HA dominated with all-era age-specific rates (15.9%; −$531; +0.010 QALYs).

**Conclusion:**

In a typical 65-year-old, aTSA dominates pyrocarbon HA. In patients aged less than 65 years, the strategies approach economic equivalence, and pyrocarbon HA is favored once aTSA revision exceeds roughly 13% at 10 years. Age-specific survivorship drives economic advantage.

Glenohumeral osteoarthritis affects 16%-20% of middle-aged and older adults on radiographic evaluation and is a leading indication for shoulder arthroplasty.^17^ Anatomic total shoulder arthroplasty (aTSA), including hybrid and all-polyethylene glenoid configurations, remains the standard of care and provides reliable pain relief and functional improvement.^8^ However, radiographic glenoid component loosening has been reported in up to 34% of patients at a long-term follow-up, and clinically significant loosening requiring revision occurs at rates of up to 1.2% per year.^18^ Pyrocarbon hemiarthroplasty (HA) has emerged as an alternative bearing surface intended to reduce the progressive glenoid wear associated with conventional metallic HA.^12^^,^^31^ It is most often proposed for younger, active patients in whom glenoid preservation is prioritized and the durability of a glenoid component is least certain.^6^^,^^12^ Revision after failure also differs with pyrocarbon HA often being converted to a total arthroplasty with glenoid bone stock largely intact, whereas revision of a failed aTSA often requires management of glenoid bone loss around a loosened component.^12^ HA with concentric glenoid reaming (ream and run) is a related glenoid-preserving option in young patients, with outcomes comparable to aTSA beyond 5 years in a matched cohort.^22^

Comparative data between pyrocarbon HA and aTSA remain limited. Although early results with pyrocarbon are encouraging, historic studies of metallic HA have been associated with less complete pain relief, progressive glenoid wear, and reduced shoulder motion compared with aTSA.^19^ Registry findings are mixed. The New Zealand registry reported similar revision rates for pyrocarbon HA and aTSA in patients aged less than 60 years and a revision rate of 0.75 per 100 component-years across 288 stemmed pyrocarbon HA at a mean age of 55 years, and the Australian registry reports 5-year cumulative revision below 5% for stemmed pyrocarbon HA in osteoarthritis.^2^^,^^14^^,^^20^ Regardless of clinical outcomes, the cost implications of these techniques have not been formally evaluated. Prior cost-effectiveness analyses in shoulder arthroplasty have focused on conventional metal HA vs. aTSA and consistently found aTSA to be both less costly and more effective over a lifetime horizon.^21^ No study has assessed the long-term costs and quality-adjusted outcomes specific to pyrocarbon HA.

This study addresses that gap using a Markov cost-utility model comparing pyrocarbon HA and aTSA over a 10-year horizon. We hypothesized that aTSA would demonstrate greater cost-effectiveness than pyrocarbon HA in a typical arthroplasty patient. Our primary aim is to define the revision-rate thresholds, cost break-even points, and quality-adjusted life year (QALY) tradeoffs that determine when each strategy becomes cost-effective and to examine how those thresholds behave in the younger patients for whom pyrocarbon HA is most often considered.

## Materials and methods

We compared the cost-effectiveness of pyrocarbon HA and aTSA using a Markov state-transition decision analytical model, a widely used framework for evaluating shoulder arthroplasty procedures.^21^ In this framework, a hypothetical cohort moves between mutually exclusive health states at fixed annual intervals according to transition probabilities and accumulates costs and QALYs for each year spent in each state.^30^ The analysis was conducted from a US hospital perspective. Costs were reported in 2026 US dollars and effectiveness was measured in QALYs. Both were discounted at 3% per year.^29^ The model was implemented in TreeAge Pro (version 2025; TreeAge Software, Williamstown, MA, USA).^32^ Key inputs are summarized in Table I. Cost-effectiveness was evaluated in a 65-year-old base-case patient over a 10-year horizon, with a secondary 5-year analysis. An age of 65 years represents a typical patient undergoing anatomic shoulder arthroplasty for primary glenohumeral osteoarthritis, recognizing that pyrocarbon HA is also proposed for younger active patients, who are addressed in scenario analyses. A willingness-to-pay threshold of $50,000 per QALY was applied, with $100,000 and $150,000 per QALY as secondary thresholds.^26^

**Table I:** Model input parameters

Parameter	Pyrocarbon HA	Hybrid aTSA	All-polyethylene aTSA	Sensitivity range	Source
Costs					
Index procedure (2026 USD)	$20,662	$20,022	$20,022	±20%	Corso et al,^10^ Lapner et al^21^
Revision to rTSA (2026 USD)	$24,794	$24,794	$24,794	±20%; scenario −25% (pyrocarbon) and +25% (aTSA)	Corso et al,^10^ Lapner et al^21^
Utilities					
Well state	0.88	0.88	0.88	0.86 to 0.92	Lapner et al^21^
Revision cycle	0.67	0.67	0.67	0.63 to 0.70	Lapner et al^21^
Post-revision state	0.88	0.88	0.88	0.70 to 0.88; scenario 0.80 and 0.70	Lapner et al,^21^ assumption
Pyrocarbon well state, scenarios	0.73, 0.82, 0.85	0.88	0.88	Fixed	Lapner et al,^21^ assumption
Revision rates, base case					
5-yr cumulative revision	6.40%	1.51%∗	3.15%∗	±50%	Boileau et al,^6^ derived
10-yr cumulative revision	10.60%	3.00%	6.20%†	±50%	Boileau et al,^6^ Codd et al,^9^ Ceccotti et al,^8^ Gill et al^16^
Revision rates, scenarios (5-yr/10-yr)					
Registry pyrocarbon, Australia (Ascend Flex PyC, OA)	4.60%/8.99%‡	—	—	Fixed	AOANJRR^2^
Registry pyrocarbon resurfacing, Australia (PyroTITAN, OA)	3.56%∗/7.00%	—	—	Fixed	AOANJRR^2^
Registry pyrocarbon, New Zealand (stemmed, OA)	3.92%§/7.69%§	—	—	Fixed	Konarski and Mohammed^20^
Registry aTSA, modern prostheses (OA)	—	3.10%/6.10%‡ (modified central peg)	4.20%/7.40% (cemented all-polyethylene)	Fixed	AOANJRR^3^
Registry aTSA, Walch B2 glenoids (OA)	—	3.80%/7.46%‡	3.80%/7.46%‡	Fixed	AOANJRR^3^
Registry aTSA, age <65, modern prostheses (OA)	—	5.50%/10.50% (95% CI, 8.3 to 13.2)	5.50%/10.50% (95% CI, 8.3 to 13.2)	Fixed	AOANJRR^3^
Registry aTSA, age 55 to 64, all eras (OA)	—	9.50%/15.90%	9.50%/15.90%	Fixed	McBride et al^24^
Registry aTSA, age <55, all eras (OA)	—	12.00%/21.10%	12.00%/21.10%	Fixed	McBride et al^24^
Model parameters					
Cycle length	1 yr	1 yr	1 yr	Fixed	
Base-case patient age	65 yrs	65 yrs	65 yrs	Fixed; scenarios <65, 55 to 64, and <55	
Time horizon	10 yrs	10 yrs	10 yrs	5 to 10 yrs	
Discount rate	3%	3%	3%	0 to 5%	Sanders et al^29^
Willingness-to-pay threshold	$50,000/QALY	$50,000/QALY	$50,000/QALY	$50,000 to $150,000/QALY	Neumann et al^26^

*HA*, hemiarthroplasty; *aTSA*, anatomic total shoulder arthroplasty; *rTSA*, reverse total shoulder arthroplasty; *OA*, osteoarthritis; *AOANJRR*, Australian Orthopaedic Association National Joint Replacement Registry; *CI*, confidence interval; *QALY*, quality-adjusted life year.

Pyrocarbon HA revision rates apply to the Tornier pyrocarbon humeral head on a stemmed humeral component unless stated.

∗ Five-year anchor derived from the 10-year value under a constant-hazard assumption.

† Extrapolated from the 9-year registry value of 5.6% under a constant-hazard assumption.

‡ Ten-year value extrapolated from the 5-year registry value under a constant-hazard assumption.

§ Converted from 0.80 revisions per 100 component-years. Hybrid aTSA base-case revision rate is the unweighted average of 2 series (range 1.8%-4.4%). In the age-specific and Walch B2 scenarios, the same rate was applied to both aTSA arms.

### Model structure

Fig. 1 illustrates the model, which includes 3 strategy-specific well states, a temporary revision state, and a permanent absorbing post-revision state. A simulated patient begins in one of 3 index procedure arms, pyrocarbon HA, hybrid aTSA, or all-polyethylene aTSA, and enters the corresponding well state after surgery. At each annual cycle, patients may remain well or transition into a one-cycle revision state representing revision to reverse total shoulder arthroplasty (rTSA), after which they enter the permanent post-revision state. All 3 well states share a common revision pathway with strategy-specific annual revision probabilities.

**Figure 1:**
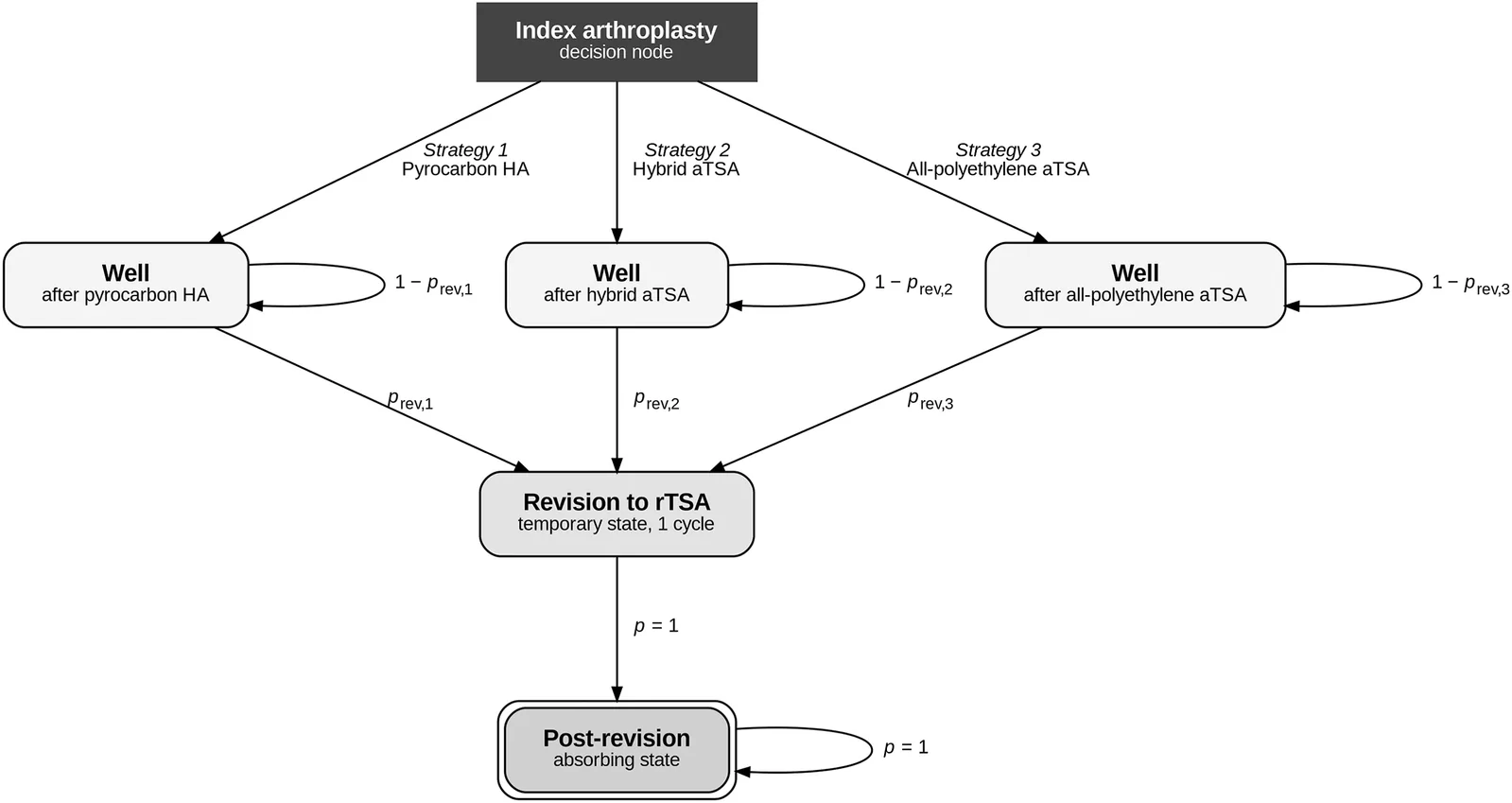
Markov state-transition model. After the index arthroplasty, patients enter the well state for their strategy. At each annual cycle, they remain well with probability 1 − p_rev,i_ or undergo revision to rTSA with the strategy-specific annual revision probability p_rev,i_, derived from the cumulative revision rates in Table I. Revision is a temporary state lasting one cycle, in which the revision cost and the revision-cycle utility are applied, after which patients enter the absorbing post-revision state. *HA*, hemiarthroplasty; *aTSA*, anatomic total shoulder arthroplasty; *rTSA*, reverse total shoulder arthroplasty.

### Costs

Procedural costs for aTSA, pyrocarbon HA, and revision to rTSA were derived from hospital billing data reported by Corso et al and Lapner et al and converted to 2026 US dollars.^10^^,^^21^ The index procedure cost was $20,022 for aTSA and $20,662 for pyrocarbon HA, the latter incorporating the Tornier pyrocarbon humeral head implant cost (Stryker, Kalamazoo, MI, USA) converted from New Zealand dollars and inflated to 2026 US dollars.^28^ Revision to rTSA was costed at $24,794 in all arms in the base case. Because revision of a failed pyrocarbon HA and of a failed aTSA may differ in complexity, scenario analyses varied the pyrocarbon revision cost by −25%, the aTSA revision cost by +25%, and both together, and a threshold analysis identified the pyrocarbon revision cost at which aTSA would lose cost-effectiveness. In a probabilistic analysis, gamma distributions were applied to costs with standard errors of 10% of the mean.

### Revision rates

Revision rates for pyrocarbon HA were derived from Boileau et al, the largest prospective series of pyrocarbon shoulder HA with a 10-year follow-up, reporting cumulative revision of 6.4% at 5 years and 10.6% at 10 years.^6^ That series comprised 103 shoulders in 96 patients with a mean age of 56 years, most of whom were working or playing sport, treated with the Tornier pyrocarbon humeral head on a convertible press-fit stem (Ascend Flex, Tornier-Stryker). Biconcave B2 and B3 glenoids were managed with corrective and concentric reaming. Hybrid aTSA revision rates were the unweighted average of Codd et al (1.8% at 10 years, Comprehensive short-stem humeral component with hybrid glenoid, Zimmer Biomet, Warsaw, IN) and Ceccotti et al (4.4% at 10 years, Comprehensive hybrid glenoid with a porous-coated central titanium post and 3 cemented peripheral pegs, 256 shoulders, mean age 69 years, Walch B1 in 29% and B2 in 28%, mean follow-up 49 months), giving a base case of 3.0% with a sensitivity band of 1.8% to 4.4% carried through all sensitivity analyses.^8^^,^^9^ All-polyethylene aTSA rates were derived from Gill et al, an Australian registry analysis of stemmed and stemless aTSA with all-polyethylene glenoids reporting 5.6% cumulative revision at 9 years, extrapolated to 6.2% at 10 years under a constant-hazard assumption.^16^

Annual transition probabilities were derived from cumulative revision anchors using *P* = 1 − (1 − R)^(1/t), where R is the cumulative revision rate at t years.^7^^,^^13^^,^^25^^,^^30^ For pyrocarbon HA, separate annual probabilities were calculated for years 1 to 5 and for years 6 to 10 from the 2 anchors. For both aTSA arms, only a late anchor was available, so a constant annual probability was applied and the 5-year values in Table I (1.5% and 3.2%) are the corresponding interpolated estimates. Registry rates reported per 100 component-years were converted to cumulative values as R = 1 − exp(−rt).^13^^,^^25^

### Scenario analyses

Because these inputs come from cohorts that differ in age, sex, glenoid morphology, implant design, and study method, 4 groups of scenario analyses were performed, drawing on the 2025 Australian registry report, which is restricted to modern prostheses and stratified by age, glenoid type, and Walch morphology for stemmed aTSA in osteoarthritis.^3^ First, registry-based pyrocarbon scenarios replaced the Boileau estimate with the Australian cumulative revision for the same stemmed construct (Ascend Flex PyC), 4.6% at 5 years extrapolated to 9.0% at 10 years, with the Australian 10-year value of 7.0% for pyrocarbon resurfacing (PyroTITAN), and with the New Zealand rate for stemmed pyrocarbon HA in osteoarthritis of 0.80 per 100 component-years, equivalent to 3.9% at 5 years and 7.7% at 10 years.^2^^,^^20^ Second, a registry-based aTSA scenario used the Australian values for cemented all-polyethylene glenoids (4.2% at 5 years and 7.4% at 10 years) and modified central-peg glenoids (3.1% at 5 years, extrapolated to 6.1% at 10 years). Third, a morphology-matched scenario applied the Australian 5-year revision for stemmed aTSA in Walch B2 glenoids (3.8%, extrapolated to 7.5% at 10 years) to both aTSA arms. Fourth, younger-patient scenarios retained the Boileau estimate, whose cohort had a mean age of 56 years, and replaced the aTSA inputs with age-specific registry values, 5.5% at 5 years and 10.5% at 10 years (95% confidence interval, 8.3%-13.2%) for modern stemmed aTSA in patients aged less than 65 years, and the all-era values reported by McBride et al for the same registry, 9.5% and 15.9% for ages 55 to 64 and 12.0% and 21.1% for ages less than 55.^3^^,^^24^ The same rates were applied to both aTSA arms in these scenarios because age-specific survivorship by glenoid type has not been reported.

### Health utility values

Health utility values were derived from Lapner et al.^21^ A base-case well-state utility of 0.88 was applied equally across all 3 arms (sensitivity range, 0.86-0.92), consistent with the median EuroQol 5-Dimension 5-Level (EQ-5D-5L) utility of 0.87 reported at follow-up in the hybrid aTSA series of Ceccotti et al.^8^ Although HA has historically been associated with modestly lower functional outcomes than aTSA, the magnitude of this difference with pyrocarbon implants is uncertain, so equal well-state utilities were used as a conservative assumption that does not favor aTSA. Patients in the revision cycle were assigned the Lapner et al utility for the revision surgical procedure state, 0.67 (range, 0.63-0.70), and patients in the post-revision state were assigned 0.88, following the Lapner et al assumption that utility after revision equals that after primary aTSA. QALY differences between arms, therefore, arise only from the proportion of patients who pass through the revision year. Because a lasting decrement after revision is plausible, scenarios applied post-revision utilities of 0.80 and 0.70. Further scenarios applied a pyrocarbon well-state utility of 0.73 from Lapner et al and intermediate values of 0.82 and 0.85, while holding aTSA at 0.88.

### Sensitivity analysis

One-way sensitivity analyses varied revision rates, costs, utilities, the discount rate, and the time horizon across the ranges in Table I. Probabilistic sensitivity analysis (PSA) used Monte Carlo simulation with 10,000 iterations to generate cost-effectiveness acceptability curves, with beta distributions for probabilities and utilities and gamma distributions for costs. Because equal utility across arms is a structural assumption of the base case, one well-state value and one revision-cycle value were drawn per iteration and applied to all arms. A secondary PSA drew utilities independently for each arm. Throughout, dominance refers to the deterministic result in which one strategy is both less costly and more effective, and probability of cost-effectiveness refers to the proportion of PSA iterations in which incremental net monetary benefit (INMB) favors that strategy. Threshold analyses identified the pyrocarbon 10-year revision rate, index cost, revision cost, and well-state utility at which pyrocarbon HA would become cost-effective against each comparator and the comparator revision rate at which aTSA would lose its advantage.

## Results

### Base-case findings

In the base case, both aTSA pathways dominated pyrocarbon HA over the 10-year horizon (Table II, Panel A). Hybrid aTSA yielded 7.726 discounted QALYs at $20,676, compared with 7.711 QALYs at $23,008 for pyrocarbon HA, an incremental cost of $2,332, and a QALY loss of 0.015 for pyrocarbon HA. All-polyethylene aTSA produced 7.720 QALYs at $21,375, an incremental cost of $1,633, and a QALY loss of 0.009 for pyrocarbon HA. At 5 years, hybrid aTSA cost $20,376 with 4.148 QALYs and all-polyethylene aTSA $20,759 with 4.145 QALYs, compared with $22,160 and 4.138 QALYs for pyrocarbon HA. The INMB of hybrid aTSA over pyrocarbon HA was $3,066 at $50,000 per QALY, $3,799 at $100,000, and $4,533 at $150,000 per QALY. For all-polyethylene aTSA, the values were $2,063, $2,494, and $2,924.

**Table II:** Base-case results, threshold, probabilistic, and scenario analyses (base-case age 65, 10-year horizon)

Panel A: Base-case cost-utility results						
Strategy	Discounted cost (2026 USD)	Discounted QALYs	10-yr revision	ΔCost vs. pyrocarbon	ΔQALYs vs. pyrocarbon	INMB at $50,000/QALY
Pyrocarbon HA	$23,008	7.711	10.60%	—	—	—
Hybrid aTSA	$20,676	7.726	3.00%	−$2,332 (dominant)	0.015	$3,066
All-polyethylene aTSA	$21,375	7.720	6.20%	−$1,633 (dominant)	0.009	$2,063

Panel B: Threshold analyses		
Threshold parameter	vs. hybrid aTSA	vs. all-polyethylene aTSA
Pyrocarbon 10-yr revision rate for cost-effectiveness	≤0.95%	≤4.16%
Pyrocarbon index cost for cost-effectiveness	≤$17,596 (↓$3,066)	≤$18,599 (↓$2,063)
Pyrocarbon well-state utility for cost-effectiveness	≥0.887	≥0.885
Pyrocarbon revision cost for cost-effectiveness	None (aTSA cost-effective at $0)	≤$2,989
Comparator 10-yr revision rate at which aTSA loses cost-effectiveness	>12.78%	>12.78%

Panel C: Probabilistic sensitivity analysis (10,000 Monte Carlo iterations)						
Comparison	ΔCost (mean, 95% CrI)	ΔQALYs (mean, 95% CrI)	% dominant	%CE at $50k	%CE at $100k	%CE at $150k
Hybrid aTSA vs. pyrocarbon, shared utility draws	−$2,309 [−$8,033 to +$3,541]	0.015 [0.005 to +0.027]	78.6%	84.3%	88.8%	91.8%
All-polyethylene aTSA vs. pyrocarbon, shared utility draws	−$1,650 [−$7,463 to +$4,139]	0.009 [−0.002 to +0.021]	67.9%	75.2%	78.8%	81.8%
Hybrid aTSA vs. pyrocarbon, independent utility draws	−$2,348 [−$8,103 to +$3,475]	0.016 [−0.347 to +0.379]	42.4%	63.0%	58.6%	57.0%
All-polyethylene aTSA vs. pyrocarbon, independent utility draws	−$1,689 [−$7,572 to +$4,273]	0.007 [−0.357 to +0.369]	37.3%	58.2%	55.4%	54.1%
aTSA vs. pyrocarbon, age <65, modern-prosthesis registry aTSA rates	−$672 [−$6,479 to +$5,117]	0.000 [−0.014 to +0.015]	35.4%	59.0%	58.9%	58.6%
aTSA vs. pyrocarbon, age 55 to 64, all-era registry aTSA rates	$550 [−$5,497 to +$6,530]	−0.010 [−0.030 to +0.007]	8.4%	37.2%	32.6%	29.1%

Panel D: Scenario analyses (deterministic, $50,000/QALY)						
Scenario	ΔCost, hybrid aTSA vs. pyrocarbon	ΔQALYs, hybrid	INMB, hybrid	ΔCost, all-polyethylene aTSA vs. pyrocarbon	ΔQALYs, all-polyethylene	INMB, all-polyethylene
Base case (age 65)	−$2,332	+0.015	$3,066	−$1,633	+0.009	$2,063
Registry pyrocarbon, Australia (9.0% at 10 yrs)	−$1,949	+0.011	$2,516	−$1,249	+0.005	$1,513
Registry pyrocarbon resurfacing, Australia (7.0% at 10 yrs)	−$1,514	+0.008	$1,892	−$814	+0.002	$890
Registry pyrocarbon, New Zealand (7.7% at 10 yrs)	−$1,664	+0.009	$2,108	−$965	+0.003	$1,106
Registry aTSA, modern prostheses (hybrid 6.1%, all-polyethylene 7.4% at 10 yrs)	−$1,654	+0.009	$2,094	−$1,357	+0.006	$1,668
Registry aTSA, Walch B2 glenoids (7.5% at 10 yrs)	−$1,359	+0.006	$1,671	−$1,359	+0.006	$1,671
Younger patient, age <65, modern-prosthesis aTSA (10.5% at 10 yrs)	−$689	+0.000	$711	−$689	+0.000	$711
Younger patient, age <65, upper confidence limit (13.2% at 10 yrs)	−$101	−0.005	−$133	−$101	−0.005	−$133
Younger patient, age 55 to 64, all-era aTSA (15.9% at 10 yrs)	+$531	−0.010	−$1,039	+$531	−0.010	−$1,039
Younger patient, age <55, all-era aTSA (21.1% at 10 yrs)	+$1,664	−0.020	−$2,662	+$1,664	−0.020	−$2,662
Younger patient, age 55 to 64, with registry pyrocarbon (9.0%)	+$915	−0.013	−$1,589	+$915	−0.013	−$1,589
Pyrocarbon revision cost −25%	−$1,746	+0.015	$2,479	−$1,046	+0.009	$1,477
aTSA revision cost +25%	−$2,169	+0.015	$2,902	−$1,295	+0.009	$1,725
Pyrocarbon revision cost −25% and aTSA revision cost +25%	−$1,582	+0.015	$2,316	−$708	+0.009	$1,138
Post-revision utility 0.80 (lasting decrement)	−$2,332	+0.041	$4,399	−$1,633	+0.026	$2,909
Post-revision utility 0.70 (lasting decrement)	−$2,332	+0.075	$6,065	−$1,633	+0.047	$3,965
Revision-cycle utility 0.88 (equal to well state)	−$2,332	+0.000	$2,332	−$1,633	+0.000	$1,633
Pyrocarbon well-state utility 0.85	−$2,332	+0.262	$15,434	−$1,633	+0.256	$14,431
Pyrocarbon well-state utility 0.82	−$2,332	+0.509	$27,802	−$1,633	+0.503	$26,799
Pyrocarbon well-state utility 0.73 (Lapner low)	−$2,332	+1.251	$64,906	−$1,633	+1.245	$63,903

*QALY*, quality-adjusted life years; *INMB*, incremental net monetary benefit; *HA*, hemiarthroplasty; *aTSA*, anatomic total shoulder arthroplasty; *CrI*, credible interval (2.5th to 97.5th percentiles of PSA draws); *%CE*, probability that aTSA is cost-effective at the stated willingness-to-pay threshold.

Negative ΔCost and positive ΔQALYs favor aTSA. A negative INMB favors pyrocarbon HA. % dominant = proportion of iterations in which aTSA was both less costly and more effective.

### Threshold analyses

For pyrocarbon HA to become cost-effective, its 10-year cumulative revision rate would need to fall from 10.6% to 1.0% against hybrid aTSA or 4.2% against all-polyethylene aTSA (Table II, Panel B). Its index cost would need to fall by $3,066 to $17,596 against hybrid aTSA or by $2,063 to $18,599 against all-polyethylene aTSA, both several times the $640 base-case cost premium of pyrocarbon HA over aTSA. Either aTSA construct lost cost-effectiveness only if its own 10-year revision rate exceeded 12.8%. Pyrocarbon HA required a well-state utility of 0.887 or higher to be cost-effective against hybrid aTSA and 0.885 against all-polyethylene aTSA, both within 0.01 of the equalized value (Fig. 2). No pyrocarbon revision cost reversed the result against hybrid aTSA, and against all-polyethylene aTSA the cost of revising a failed pyrocarbon HA would need to fall below $2,989, about one-eighth of the base-case value.

**Figure 2:**
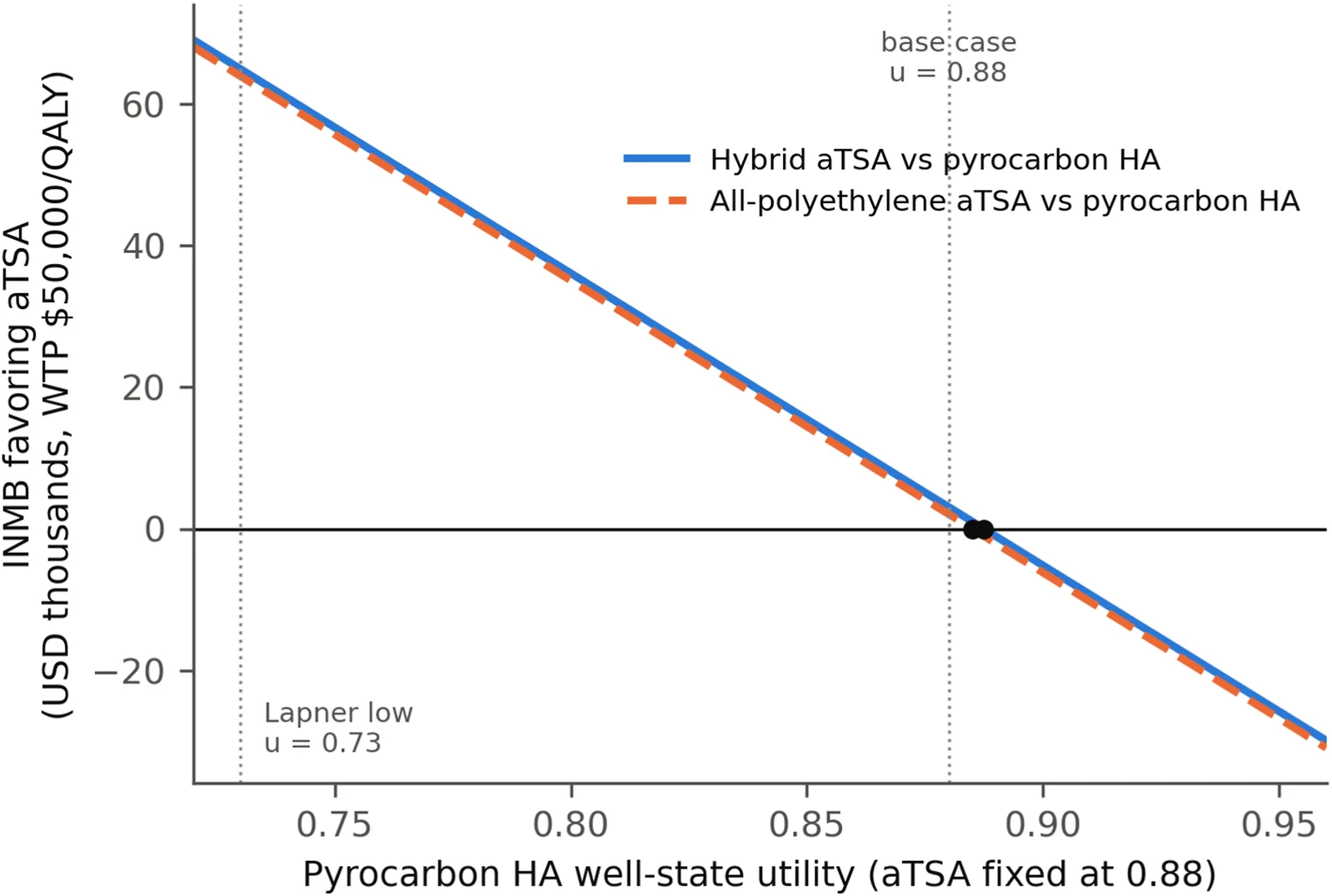
Pyrocarbon HA well-state utility threshold analysis with aTSA well-state utility fixed at 0.88. Incremental net monetary benefit favoring aTSA at a willingness to pay $50,000 per QALY is shown for hybrid aTSA (solid line) and all-polyethylene aTSA (dashed line). Points mark the utility at which INMB crosses zero. *HA*, hemiarthroplasty; *aTSA*, anatomic total shoulder arthroplasty; *INMB*, incremental net monetary benefit; *QALY*, quality-adjusted life year.

### Scenario analyses

Both aTSA arms remained dominant with every registry-based pyrocarbon rate (Table II, Panel D). The INMB of hybrid and all-polyethylene aTSA was $2,516 and $1,513 at the Australian value of 9.0%, $1,892 and $890 at the resurfacing value of 7.0%, and $2,108 and $1,106 at the New Zealand value of 7.7%. With modern-prosthesis registry aTSA inputs, hybrid aTSA (central-peg glenoid, 6.1%) had an INMB of $2,094 and all-polyethylene aTSA (7.4%) $1,668. In the Walch B2-matched scenario (aTSA 7.5%), aTSA remained dominant (−$1,359, +0.006 QALYs, INMB $1,671).

In the younger-patient scenarios, the result moved to the threshold and beyond it. With modern-prosthesis registry, aTSA rates for patients aged less than 65 years (10.5% at 10 years), aTSA remained less costly by $689 with a QALY difference of less than 0.001, an INMB of $711, and a 59.0% probability of cost-effectiveness. At the upper confidence limit of that rate (13.2%), the INMB was −$133 and pyrocarbon HA was favored. With the all-era registry rates for ages 55 to 64 (15.9%), aTSA costs $23,539 and yielded 7.701 QALYs against $23,008 and 7.711 QALYs for pyrocarbon HA, so pyrocarbon HA dominated by $531 and 0.010 QALYs (INMB favoring aTSA −$1,039) and was cost-effective in 62.8% of iterations. With the rates for ages less than 55 (21.1%), pyrocarbon HA dominated by $1,664 and 0.020 QALYs (INMB −$2,662), and with the registry-based pyrocarbon rate in the 55 to 64 scenario, it dominated by $915 and 0.013 QALYs. Figure 3 shows INMB as a continuous function of the aTSA 10-year revision rate. The crossover occurs at 12.8% with either pyrocarbon estimate. The modern-prosthesis rate for patients aged less than 65 years lies just below it and the all-era age-specific rates lie beyond it.

**Figure 3:**
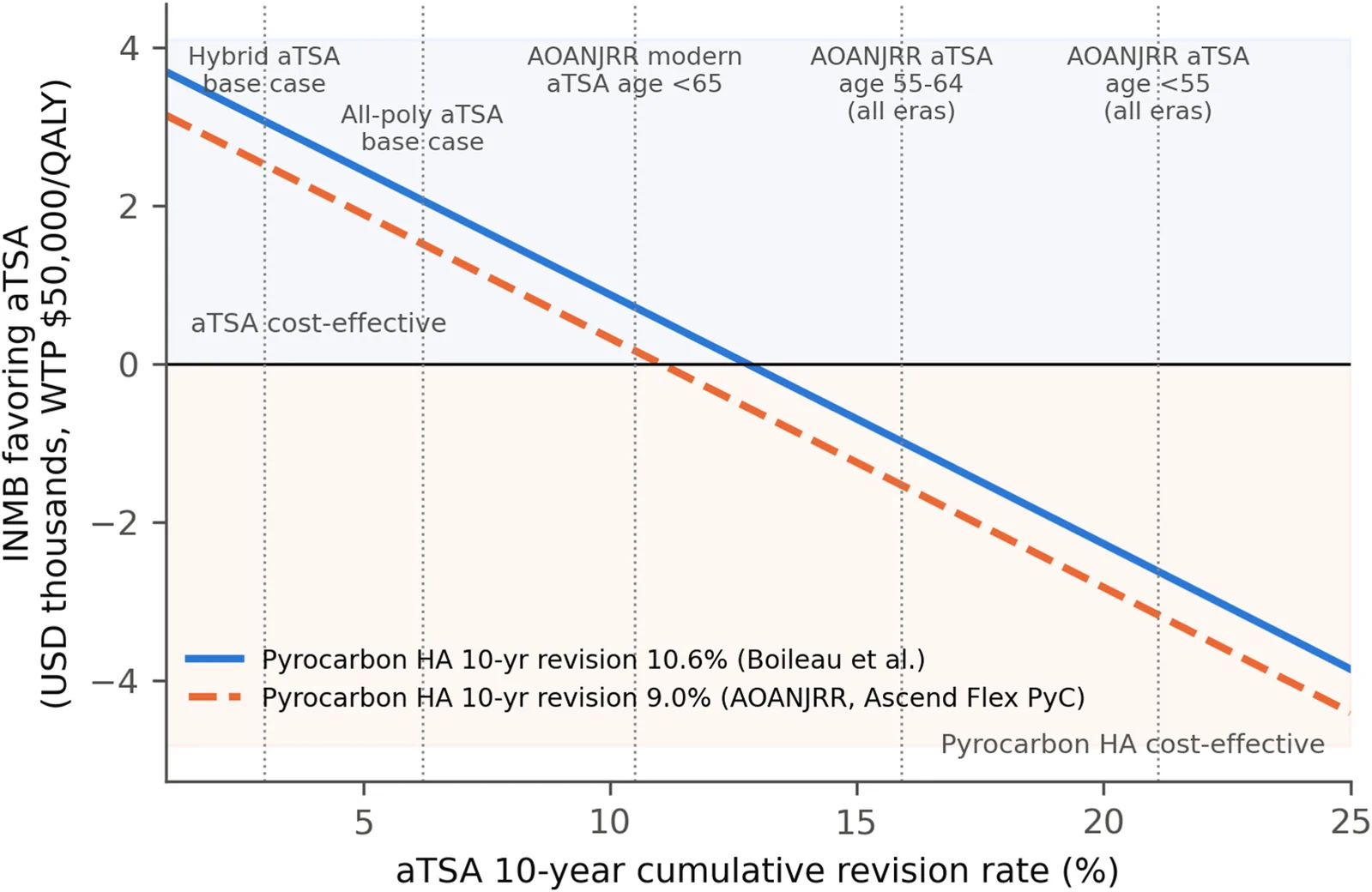
INMB favoring hybrid aTSA at $50,000 per QALY as a function of the aTSA 10-year cumulative revision rate, shown for the prospective series pyrocarbon revision estimate (solid line) and the Australian registry estimate (dashed line). Vertical reference lines mark the base-case aTSA inputs, the Australian registry modern-prosthesis value for patients aged less than 65 years, and the all-era registry values for patients aged 55-64 and less than 55 years. *aTSA*, anatomic total shoulder arthroplasty; *AOANJRR*, Australian Orthopaedic Association National Joint Replacement Registry; *HA*, hemiarthroplasty; *INMB*, incremental net monetary benefit; *QALY*, quality-adjusted life year.

Reducing the pyrocarbon revision cost by 25% lowered the INMB of hybrid and all-polyethylene aTSA to $2,479 and $1,477, raising the aTSA revision cost by 25% gave $2,902 and $1,725, and both changes together gave $2,316 and $1,138. Both aTSA arms remained dominant in every revision-cost scenario. Applying a lasting post-revision utility decrement increased the aTSA advantage, with INMB values of $4,399 and $2,909 at a post-revision utility of 0.80 and $6,065 and $3,965 at 0.70. Setting the revision-cycle utility equal to the well state removed all QALY differences, leaving aTSA less costly by $2,332 and $1,633. With pyrocarbon utility at 0.73, the INMB of hybrid aTSA increased to $64,906 at $50,000 per QALY, and with pyrocarbon utility at 0.85 and 0.82, it was $15,434 and $27,802 (all-polyethylene aTSA $14,431 and $26,799).

### Sensitivity analysis

The most influential parameters in one-way analysis were the index procedure costs of both strategies, followed by the pyrocarbon 10-year revision rate and the post-revision utility (Fig. 4). Only the ±20% index cost ranges crossed zero, and no revision-rate or utility variation within the tested ranges reversed the dominance of either aTSA strategy. In PSA with shared utility draws, hybrid aTSA was cost-effective in 84.3% of iterations at $50,000 per QALY, 88.8% at $100,000, and 91.8% at $150,000 and was dominant in 78.6% (Table II, Panel C, Fig. 5). All-polyethylene aTSA was cost-effective in 75.2%, 78.8%, and 81.8% of iterations and dominant in 67.9%. The mean incremental cost of hybrid aTSA vs. pyrocarbon HA was −$2,309 (95% credible interval, −$8,033 to +$3,541) with a mean QALY gain of 0.015 (+0.005 to +0.027). For all-polyethylene aTSA, the values were −$1,650 (−$7,463 to +$4,139) and 0.009 (−0.002 to +0.021). With utilities drawn independently for each arm, the probability of cost-effectiveness at $50,000 per QALY fell to 63.0% for hybrid and 58.2% for all-polyethylene aTSA, because independent draws add QALY noise unrelated to revision burden.

**Figure 4:**
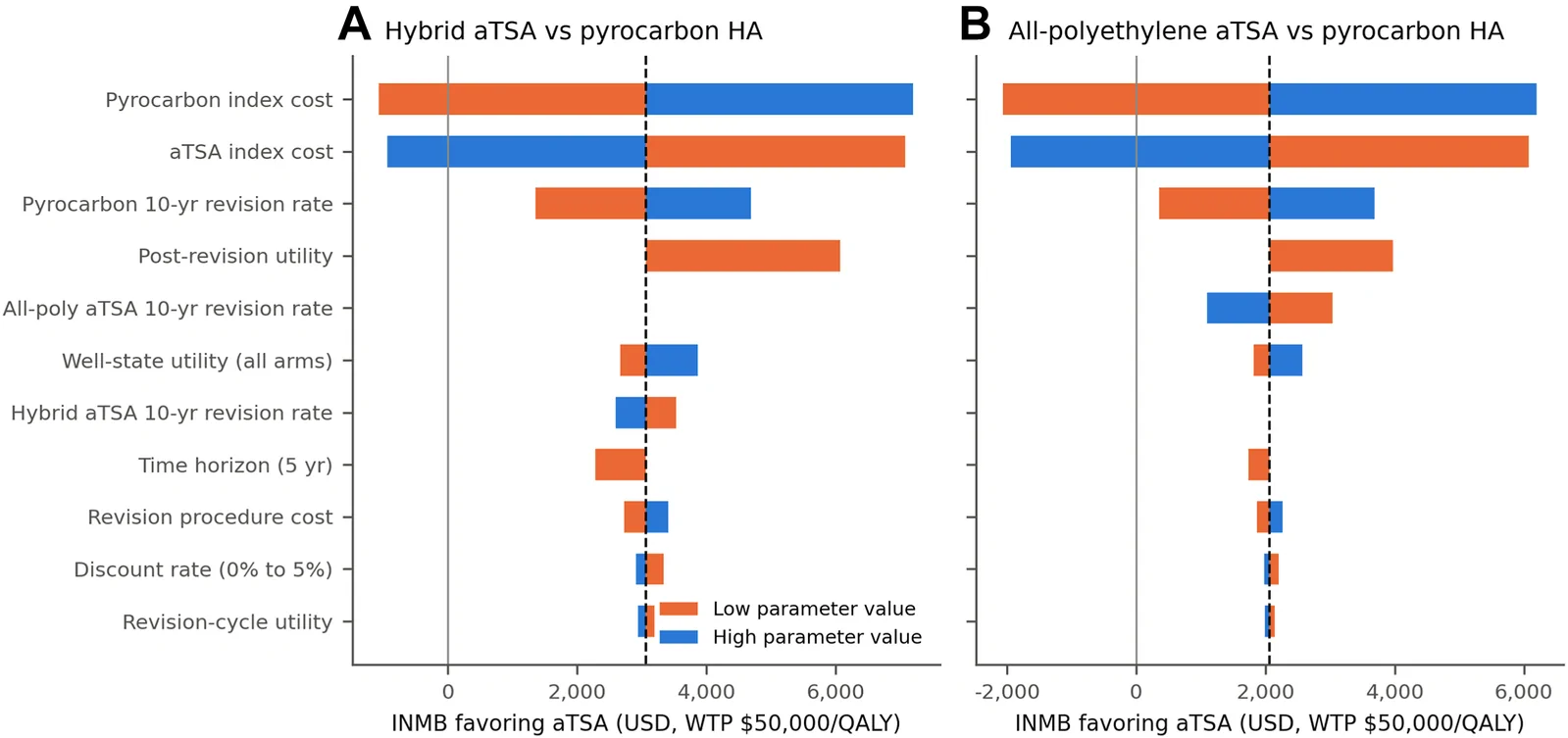
One-way sensitivity analysis. Tornado diagrams of INMB favoring aTSA at $50,000 per QALY for (**A**) hybrid aTSA vs. pyrocarbon HA and (**B**) all-polyethylene aTSA vs. pyrocarbon HA. The dashed vertical line marks the base-case INMB and the gray line marks zero. *aTSA*, anatomic total shoulder arthroplasty; *HA*, hemiarthroplasty; *INMB*, incremental net monetary benefit; *QALY*, quality-adjusted life year.

**Figure 5:**
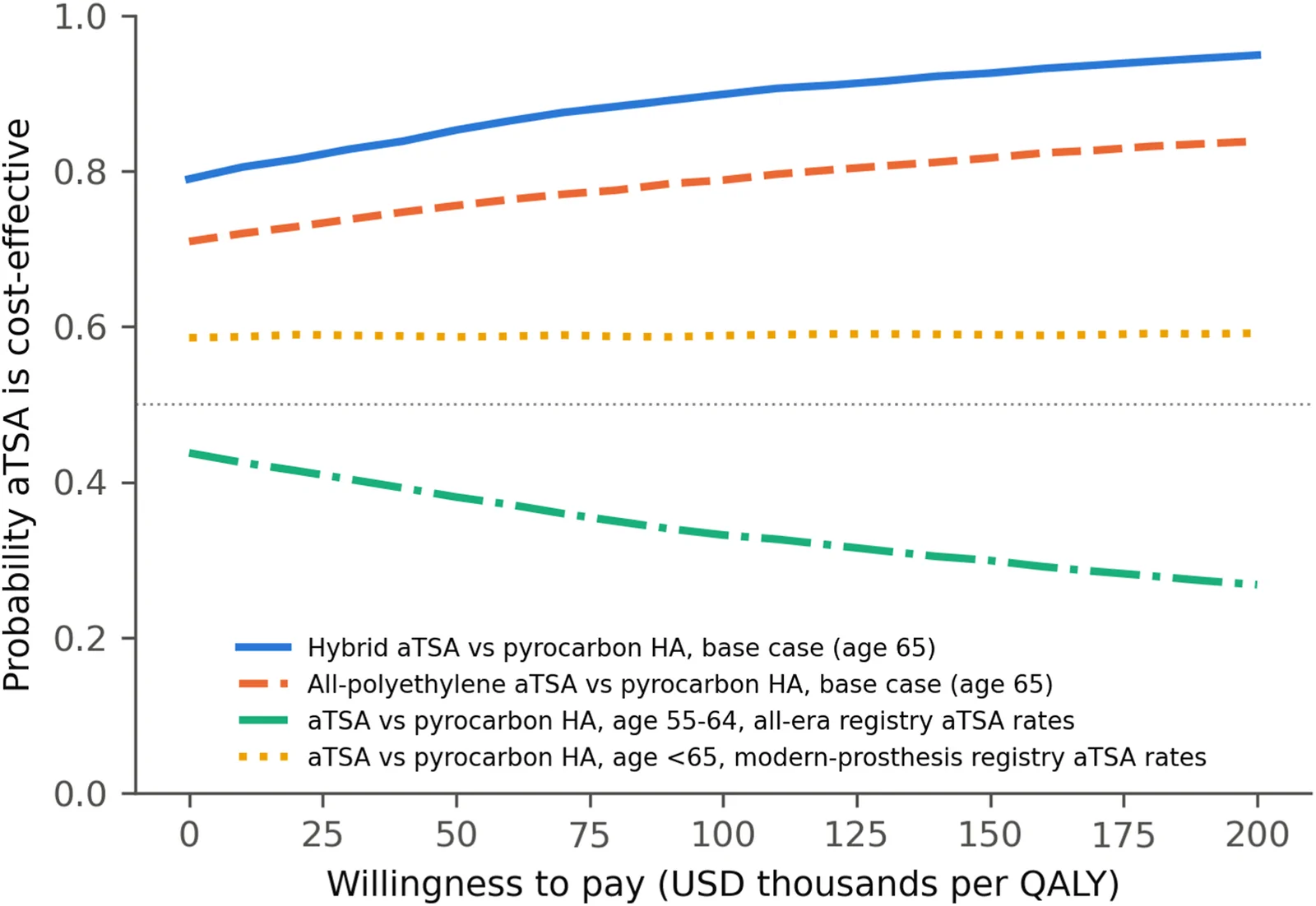
Cost-effectiveness acceptability curves from probabilistic sensitivity analysis showing the probability that aTSA is cost-effective across willingness-to-pay thresholds for the base case (age 65) and the younger-patient scenarios using modern-prosthesis registry aTSA rates (aged less than 65 years) and all-era registry rates (aged 55-64 years). *aTSA*, anatomic total shoulder arthroplasty; *HA*, hemiarthroplasty; *QALY*, quality-adjusted life year.

## Discussion

Pyrocarbon HA has entered practice on the strength of biomechanical arguments and short-term series, but its economic standing relative to modern aTSA has not been tested. This analysis finds that the answer depends on the patient. In a 65-year-old with primary glenohumeral osteoarthritis, aTSA with either a hybrid or an all-polyethylene glenoid was less costly and more effective than pyrocarbon HA over 10 years and remained cost-effective in most probabilistic simulations. With well-state utilities equalized, revision burden is the only source of the QALY difference, and the pyrocarbon revision rate from the largest prospective series is roughly 3 and a half times that of contemporary hybrid aTSA and nearly twice that of all-polyethylene aTSA. The index cost premium of pyrocarbon HA is small, and the price reduction that would make it cost-effective is several times that premium. The result held with each of the 3 registry pyrocarbon values, with modern-prosthesis registry aTSA values, and with aTSA revision restricted to B2 glenoids. In younger patients, the picture changes. The modern-prosthesis registry rate for aTSA in patients aged less than 65 years sits just below the comparator threshold identified here, and the all-era age-specific rates sit beyond it, where pyrocarbon HA becomes the dominant strategy.

Glenoid loosening has historically accounted for a large share of aTSA complications, with radiolucent lines in about a third of early cemented keeled implants.^5^^,^^33^ Design has since moved to pegged all-polyethylene components with refined cementing and polyethylene manufacturing,^11^ which show high clinical survivorship in small series, although radiographic survival is lower than clinical survival and radiolucency remains common.^23^^,^^27^ Hybrid constructs with a porous metal central post were developed to address late loosening, and the 2 hybrid series used here report 10-year revision in less than one shoulder in 20.^1^^,^^8^^,^^9^ These implants are not without concern. A multicenter computed tomography study of a hybrid glenoid with a porous titanium lower peg found complete integration of that peg in about two-thirds of shoulders and no integration at either peg in about one in 11 at 12 to 24 months, although none had been revised.^4^

Pyrocarbon HA carries well-described biomechanical advantages. Its elastic modulus is close to that of cortical bone, which lowers stress at the bearing interface and may slow glenoid erosion, and because no glenoid component is implanted, bone stock is preserved and later conversion to a total arthroplasty is simpler than revision of a loosened glenoid component.^12^ Pyrolytic carbon surfaces also show favorable wear characteristics and in vitro cartilaginous matrix formation.^28^^,^^31^ The economic case depends on whether these advantages translate into survivorship and function that compete with modern aTSA in the same patients. At the base-case rates, this has not been demonstrated, and the shared revision pathway does not explain the result, since hybrid aTSA remained cost-effective in the 65-year-old even when revision of a failed pyrocarbon HA was made free. The Boileau series identified humeral head oversizing as the main risk factor for revision, and both registry estimates for the same construct are already lower than the prospective value. Should revision rates fall below the thresholds identified here, or should the implant be priced below aTSA as manufacturing scales, pyrocarbon's economic profile would shift materially.

The central finding is that age determines the economic answer. In patients aged less than 65 years with modern prostheses, aTSA and pyrocarbon HA are within a few hundred dollars and a thousandth of a QALY of each other, and the upper confidence limit of the registry aTSA revision rate crosses the threshold at which pyrocarbon HA becomes the better value. With the registry rates for ages 55 to 64 and under 55 across all prosthesis generations, pyrocarbon HA dominates. Single-center data point the same way. In patients aged less than 60 years with all-polyethylene glenoids, Gauci et al reported revision in roughly one in 5 shoulders at a mean of 10 years.^15^ This is the comparison that matters clinically, because the pyrocarbon cohort that supplied the revision input had a mean age of 56 years and most of its patients were working or playing sport. In that population, the modern-prosthesis registry value is the relevant benchmark, and it leaves the 2 strategies close to economic equivalence, so surgeon and patient preference for glenoid preservation can reasonably decide the choice. Glenoid morphology does not appear to drive the result. Registry aTSA revision at 5 years was no higher in B2 than in A1 or B1 glenoids, and aTSA remained cost-effective against the predominantly B2 and B3 pyrocarbon cohort at the B2 rate.

From a health system perspective, these findings support aTSA as the default cost-effective strategy in the typical arthroplasty patient. They should not be read as disqualifying pyrocarbon HA, and in younger patients, the 2 strategies are economically close. The thresholds are performance targets, revision rate, and pricing benchmarks, against which future pyrocarbon survivorship data from registries, prospective series, and implant negotiations can be judged. Prior cost-utility analyses have shown that aTSA remains dominant whenever its revision rate is lower than the comparator's, underscoring survivorship as the key determinant in shoulder arthroplasty models.^21^ The present analysis shows where that balance tips. Long-term data for modern hybrid constructs in younger patients remain limited, and much of the existing evidence reflects older designs.^15^ In the Australian registry, patients aged less than 65 years undergoing modern stemmed aTSA for osteoarthritis experience about two-thirds more cumulative revision at 10 years than those aged 65 years and more.^3^

This study has several limitations. First, and most important, the revision inputs come from cohorts that are not comparable in age, sex, activity, or glenoid morphology. The pyrocarbon estimate rests on a single prospective series of young, active patients with predominantly B2 and B3 glenoids, and the aTSA estimates come from older patients and unselected registry populations. Registry aTSA revision by Walch type is available only to 5 years and excludes B3 glenoids, so the morphology-matched scenario is partial. The registry-based and younger-patient scenarios address the mismatch in part, and the result moves from clear aTSA dominance to near equivalence between them, which is itself the main message. The cost of augmented or offset implants for severe deformity was not modeled, and registry data show higher revision with augmented glenoids. Second, pyrocarbon revision rates come from a limited number of long-term series in which each revision in a small cohort has a large effect on survivorship, and the durability of modern pyrocarbon implants remains incompletely defined. Third, the analysis concerns one pyrocarbon implant, the Tornier pyrocarbon humeral head on a stemmed humeral component. Given the variation in implant design, these results should not be assumed to apply to other pyrocarbon designs such as resurfacing implants or to glenoid components other than those in the source series. Fourth, constant annual transition probabilities within each period may not capture late failure. Fifth, well-state utilities were equalized because comparative quality-of-life data between pyrocarbon HA and aTSA do not exist, and the assumption that utility after revision equals that after primary aTSA follows Lapner et al but is untested. The utility threshold shows that a well-state advantage of less than 0.01 for pyrocarbon HA would offset its revision burden, so this assumption matters. Sixth, the base-case age and 10-year horizon do not reflect the lifetime experience of younger patients, and a lifetime horizon could not be modeled because long-term comparative data in this population are not available, so background mortality was also omitted. Seventh, the younger-patient scenarios applied the same registry aTSA rates to both aTSA arms because age-specific data by glenoid type are lacking, and the all-era rates include metal-backed designs no longer in use. The model does not account for activity level, bone quality, or surgeon preference. Finally, the analysis was conducted from a US hospital perspective with some inputs converted from Canadian and New Zealand sources. Cost structures differ across health systems, so the absolute values are not generalizable outside the United States, although the revision-rate thresholds, which do not depend on price level, are.

## Conclusion

In a typical 65-year-old with primary glenohumeral osteoarthritis, both hybrid and all-polyethylene aTSA dominate Tornier pyrocarbon HA at currently published revision rates across 5-year and 10-year horizons. For pyrocarbon HA to become competitive in this patient, its 10-year cumulative revision rate would need to fall below 1.0% or 4.2%, or its index cost would need to decrease by $3,066 or $2,063. Both aTSA constructs lose that advantage when their own 10-year revision rate exceeds 12.8%. Modern-prosthesis registry rates for patients aged less than 65 years fall just short of that level, leaving the 2 strategies close to economic equivalence, and all-era age-specific rates exceed it, where pyrocarbon HA dominates. The economic choice is, therefore, determined by age-specific survivorship rather than by implant price, and it rests on cohorts that differ in age and glenoid morphology, which limits its precision. These thresholds define the performance targets that prospective survivorship data and implant pricing must meet for pyrocarbon HA's glenoid-preserving advantages to translate into economic competitiveness with modern aTSA.

## Disclaimers:

Funding: No funding was disclosed by the authors.

Conflicts of interest: Joseph A. Abboud reports that he has received royalties from Osteocentric Technologies, Enovis, Zimmer Biomet, Stryker, and Globus Medical, Inc.; holds stocks in Shoulder Jam, Aevumed, Oberd, OTS Medical, Orthobullets, Atreon, and Restore 3D; has received research support as a PI from Enovis and Arthrex; has received royalties and financial or material support from Wolters Kluwer, Slack Orthopaedics, and Elsevier; and is a board member/committee appointments for a society of American Shoulder and Elbow Surgeons, Mid Atlantic Shoulder and Elbow Society, Shoulder 360, and Pacira.

Grant E. Garrigues reports that he has received board membership from ASES; is in the editorial board of JSES and Techniques in Orthopaedics; is a consultant for Bioventus, Tornier, Mitek, and DJ Orthopaedics; has received royalties from Tornier and DJ Orthopaedics; has received other financial support from Arthrex, Inc., Zimmer, and SouthTech; holds stocks in Genesys and ROM 3.

Robert J. Gillespie reports that he holds stock or stock options in Aevumed and Genesis Innovation Group; is a paid consultant for DJ Orthopaedics, Shoulder Innovations, and Stryker; is a paid presenter or speaker for DJ Orthopaedics and Stryker; and has received intellectual property royalties from Shoulder Innovations.

Thibault Lafosse reports that he has a relationship with Smith and Nephew that includes consulting or advisory; has a relationship with DePuy Synthes Mitek Sports Medicine that includes consulting or advisory; has a relationship with Zimmer Biomet Holdings Inc. that includes consulting or advisory; and has a relationship with Stryker Orthopaedics that includes consulting or advisory.

William N. Levine reports that he has received royalties or licenses and consulting fees from Zimmer Biomet Holdings, Inc.; is the past president of American Shoulder and Elbow Surgeons; and is in the editorial board of Journal of American Academy of Orthopaedic Surgeons and Journal of Shoulder and Elbow Surgery.

Hafiz F. Kassam reports that he is a consultant for Zimmer Biomet.

Any additional authors, their immediate families, and any research foundations with which they are affiliated have not received any financial payments or other benefits from any commercial entity related to the subject of this article.

## Ethics declaration

The authors have NOT obtained informed consent from participants or their legal representatives. Using published data.

This study was performed in compliance with relevant laws, regulatory frameworks and guidelines where the research took place. Ethics committee approval was not required under relevant laws and institutional guidelines. Using published data.
